# Pulmonary infection and baseline mRS scores predict poor prognosis in anti-GABA_B_R encephalitis

**DOI:** 10.3389/fimmu.2022.918064

**Published:** 2022-08-25

**Authors:** Junqing Ding, Dingkang Xu, Jie Lv, Tianwen Wu, Jinghong Li, Mi Tian, Yajun Lian

**Affiliations:** ^1^ Department of Neurology, The First Affiliated Hospital of Zhengzhou University, Zhengzhou, China; ^2^ Department of Neurosurgery, Beijing Hospital, National Center of Gerontology, Institute of Geriatric Medicine, Chinese Academy of Medical Sciences, Beijing, China; ^3^ Graduate School of Peking Union Medical College, Beijing, China; ^4^ Department of Nephrology and Rheumatology, Dongzhimen Hospital, The First Affiliated Hospital of Beijing University of Chinese Medicine, Beijing, China

**Keywords:** anti-gamma-aminobutyric-acid B receptor (anti-GABABR) encephalitis, Baseline mRS score, pulmonary infection, prognosis, absolute lymphocyte count (ALC), Hyponatremia

## Abstract

**Purpose:**

Anti-gamma-aminobutyric-acid type B receptor (anti-GABA_B_R) encephalitis is a rare autoimmune condition caused by the presence of GABA_B_R antibodies in the limbic system. However, its clinical features and prognostic factors are poorly understood. In this study, we aimed to explore factors that affect the response to first-line treatment in patients with anti-GABA_B_R encephalitis.

**Methods:**

Thirty-four patients with an initial diagnosis of anti-GABA_B_R encephalitis were retrospectively enrolled from December 2015 to June 2021. Clinical features and experimental data recorded within 24 h of admission were extracted from the patients’ medical records. The modified Rankin Scale (mRS) was utilized to assess disease severity at admission and functional recovery after immunotherapy. Independent prognostic factors were determined by ordinal logistic regression analysis.

**Results:**

Of the 34 anti-GABA_B_R encephalitis patients, 12 (35%) presented with cancer; all of these patients had lung cancer. According to multivariate regression analysis, the cancer group exhibited a decrease in the peripheral blood absolute lymphocyte count (ALC) (odds ratio [OR]: 0.063, 95% confidence interval [CI]: 0.006-0.639, P=0.019) and hyponatremia (OR: 9.268, 95% CI: 1.054-81.502, 0.045). In addition, the neutrophil/lymphocyte ratio (NLR), monocyte/lymphocyte ratio (MLR) and platelet/lymphocyte ratio (PLR) did not significantly differ according to mRS scores in patients receiving first-line treatment. No patients with mild or moderate mRS scores (0-2) at admission developed symptoms after treatment; in contrast, only 11 patients with a severe mRS scores (≥3, 11/18) experienced symptom alleviation. Ordinal regression analysis indicated that worse prognosis was associated with pulmonary infection (OR=9.885, 95% CI: 1.106-88.323, P=0.040) and baseline mRS scores (OR= 24.047, 95% CI: 3.294-175.739, P=0.002) in the adjusted model.

**Conclusion:**

Our findings demonstrate that pulmonary infection and baseline mRS scores are independent risk factors for poor prognosis in patients with anti-GABA_B_R encephalitis after first-line treatment. ALC and hyponatremia are potential biomarkers for anti-GABA_B_R encephalitis cases accompanied by lung cancer.

## Introduction

Autoimmune encephalitis (AE) is an inflammation of the central nervous system (CNS) triggered by immune system attack of the CNS and the production of aberrant pathogenic autoantibodies (1). AE can be divided into various types according to the production of autoantibodies against neuronal cell surface or synaptic proteins. Anti-GABA_B_R encephalitis is the third most frequent AE after anti-N-methyl-D-aspartate receptor (anti-NMDAR) encephalitis and anti-leucine-rich, glioma-inactivated 1 receptor (anti-LGI1) encephalitis. However, anti-GABA_B_R encephalitis is relatively rare, accounting for approximately 5% of AE cases (2). Anti-GABA_B_R encephalitis, first reported by Lancaster et al. in 2010 (3), is characterized by the presence of limbic encephalitis, including the acute or subacute onset of prominent seizures, cognitive dysfunction, and psychiatric behavior (4). Approximately 50% of these patients harbor an underlying cancer, particularly small-cell lung cancer (SCLC) or a pulmonary neuroendocrine tumor (5–7); therefore, anti-GABA_B_R encephalitis is also known as paraneoplastic limbic encephalitis (PLE).

As anti-GABA_B_R encephalitis is chiefly mediated by humoral immunity, management of this condition focuses on immunotherapy and the detection and removal of tumors (8). First-line treatments include steroids, intravenous immunoglobulin (IVIG), and plasma exchange (PLEX), either alone or in combination; rituximab, cyclophosphamide, and bortezomib comprise second-line immunotherapies (9). Patients usually respond well to immunotherapy, which alleviates 70%–83.3% of neurological symptoms (10), and treatment of the associated cancer (11).

In general, the interaction between peripheral immune cell ratios and clinical outcomes in AE patients has attracted significant attention. Recent studies of AE have found that a high NLR significantly correlates with long-term functional disability, as measured by the mRS scores, and a reduced response to first-line immunotherapy (12, 13). James Broadley et al. (14) showed that a high NLR was associated with failure of first-line treatment but that a high MLR was not associated with AE patient prognosis. The PLR has recently been associated with prognosis in various diseases, such as lung cancer, affective disorders and diabetic kidney disease (15–17). However, no studies have examined PLR as a prognostic biomarker in AE.

Previous studies of anti-GABA_B_R encephalitis have mostly been descriptive, utilizing individual cases or small samples and evaluating clinical symptoms and long-term prognosis. No study has focused on predictive factors for evaluating the use of immunotherapy as first-line treatment. In this study, data from 34 patients admitted to our hospital with an initial diagnosis of anti-GABA_B_R encephalitis were analyzed to explore the clinical characteristics of anti-GABA_B_R encephalitis and to identify factors that predicted poor prognosis after first-line treatment, allowing combined first-line immunotherapy and second-line immunotherapy to be administered in a timely manner.

## Methods

### Participants

This retrospective study was approved by the Ethics Committee of the First Affiliated Hospital of Zhengzhou University in accordance with Helsinki declaration. The patients/proxy provided written informed consent prior to participation in this study. Thirty-four patients who were admitted to the First Affiliated Hospital of Zhengzhou University from December 2015 to June 2021 with an initial diagnosis of anti-GABA_B_R encephalitis were selected for inclusion. The diagnosis was based on the consensus for diagnosis and treatment of AE proposed by Chinese experts in 2017. All included patients met the following diagnostic criteria for anti-GABA_B_R encephalitis: (1) clinical manifestations of limbic encephalitis, such as the acute or subacute onset of prominent seizures, cognitive dysfunction, and psychiatric behavior; (2) positive results on tests for anti-GABA_B_R antibodies in cerebrospinal fluid (CSF) and/or serum; and (3) received first-line treatment. The exclusion criteria were as follows: (1) anti-GABA_B_R encephalitis was confirmed and treated before admission; (2) diagnosis of infectious, toxic, or metabolic encephalopathy and/or another nervous system disease prior to the onset of anti-GABA_B_R encephalitis; (3) incomplete clinical data; or (4) loss at follow-up. For each patient, follow-up evaluations were conducted by telephone or outpatient interviews for at least 6 months.

### Data collection

The following basic clinical data were collected: demographic characteristics (age and sex), interval from onset to admission, clinical manifestations (prodrome, initial symptoms, and primary clinical manifestations), immunotherapy latency, treatment methods, admission to the ICU, and complications (pulmonary infection, central hypoventilation, hypoproteinemia, and hyponatremia). We defined immunotherapy latency as the interval from onset to the initiation of immunotherapy. Pulmonary infection was diagnosed by respiratory physicians according to relevant criteria.

The results of laboratory tests and imaging examinations were also extracted from medical records and electronic databases for review. Abnormal cranial magnetic resonance imaging (MRI) results were confirmed as consistent with neuroinflammation (18), including T2-weighted fluid-attenuated inversion recovery (FLAIR) hyperintensities on one or both sides of the mesial temporal lobes (hippocampus and amygdala). We determined the CSF pressure, white blood cell (WBC) count, lymphocyte ratios, total protein, and autoantibody results from serum and CSF samples based on the first lumbar puncture after admission. Immunoglobulin anti-GABA_B_R antibodies in the CSF were detected by cell-based assays (CBAs) in all patients. To prevent potential impacts on peripheral immune cell counts, we excluded patients with systemic infections or who underwent immunotherapy. In addition, we obtained the total WBC count, platelet count (PLT), absolute neutrophil count (ANC), absolute lymphocyte count (ALC) and absolute monocyte count (AMC) from the patient’s first full blood analysis within 24 h of admission. The NLR was calculated as the ratio of ANC to ALC; the MLR and PLR were calculated in a similar manner. In this study, all patients received examinations that screened for tumors, including computed tomography (CT) scans of the thorax, and ultrasounds of the abdomen, pelvic area and reproductive regions during hospitalization.

### Disease prognosis evaluation

The mRS was used to evaluate the neurological function of the patient at the time of admission, in the first 4 weeks of treatment (19), and during the follow-up period. The mRS scores include 6 categories (20). Patients were divided into the mild or moderate group (0-2) and severe group (3-6) according to their mRS scores at admission.

### Statistical analysis

Missing data were imputed using multiple imputation methods. The Shapiro–Wilk test was applied to assess the distribution of data. Continuous variables with a normal distribution are presented as the mean ± standard deviation. For data with a skewed distribution, the median (1^st^ quartile, 3^rd^ quartile) was utilized to describe their features, and Kruskal–Wallis tests were employed for comparisons. Categorical variables are presented as frequencies (proportions), and Fisher’s exact tests were applied for comparisons. Parameters with P< 0.05 in the univariate analysis were included in the ordinal logistic regression analysis to estimate the effect of treatment on the full range of the mRS scores. Tolerance and the variance inflation factor (VIF) were used to examine multicollinearity. ALC, CSF WBC count, and the PLR were included in binary logistic regression analysis with the outcome of tumor presentation; the presence of psychiatric behavior was ignored due to its extreme effect. Ordinal logistic regression was performed to investigate risk factors. Model 1 included mRS score at admission, hospital stay, psychiatric behavior, tumor presentation, central hypoventilation, pneumonia, hypoproteinemia and mRS score after immunotherapy. To further test the stability of the model, age and sex were included as covariates in Model 2, while hospital stay was not adjusted for. A P value < 0.05 was considered statistically significant. Descriptive analysis of the baseline and univariate analyses was performed using IBM SPSS version 25.0 for Windows.

## Results

### Clinical characteristics

In total, 42 potential patients were screened; of these, 34 met the inclusion criteria. The baseline clinical features of the study population are shown in **Table 1**. The median age of the patients was 62.5 (15-82) years old, and the sample included 26 (76.50%) men and 8 (23.50%) women. All patients had an acute or subacute onset, and the median time from onset to admission was 10 (1-180) days. 13(38.2%) exhibited prodromal symptoms, with 6 having a fever and 5 having headaches. Other prodromal symptoms included dizziness, fatigue, vomiting, diarrhea, and sore throat. The most common initial symptom was seizure (26/34, 76.5%). 3 (8.8%) initially experienced behavioral changes, and 3 (8.8%) patients presented with memory deficits as the initial symptom. The primary clinical manifestations included seizure (n = 30, 88.2%), psychiatric behavior (n = 23, 67.6%), cognitive dysfunction (n = 23, 67.6%), Consciousness declination (n = 12, 35.3%), sleep disorders (n=10, 29.4%), movement disorders (n = 5, 14.7%), speech dysfunction (n = 4, 11.8%) and autonomic dysfunction (n = 4, 11.8%). Among these patients, 12 (35.3%) were admitted to the ICU for supportive treatment. Regarding complications, half of the patients in this cohort (n = 17, 50%) had pulmonary infections, followed by those with hypoproteinemia (n = 10, 29.4%), hyponatremia (n = 8, 23.5%) and central hypoventilation (n = 5, 14.7%).

**Table 1:** Patient characteristics in the cancer and noncancer groups.

	Total	Cancer	Noncancer	P value	OR (95% CI), P
**Variable**	34	12	22	–	
**Males, n (%)**	26	10	16	0.548	
**Age, median (IQR), years**	62.5 (54.75-65.25)	64.5 (59.25-66.5)	59 (48-64.5)	0.784	
**mRS score at admission, mild or moderate, n (%)**	17	4	13	0.102	
**Symptoms**					
Psychiatric behavior, n (%)	23	12	11	0.003^*^	
Seizure, n (%)	30	11	19	1.000	
Consciousness declination, n (%)	12	7	5	0.062	
Cognitive dysfunction, n (%)	23	8	15	1.000	
Movement disorder, n (%)	5	2	3	1.000	
Speech dysfunction, n (%)	4	0	4	0.273	
Sleep disorder, n (%)	10	5	5	0.271	
Autonomic dysfunction, n (%)	4	2	2	0.602	
Prodromal symptoms, n (%)	11	6	5	0.138	
**ICU admission, n (%)**	12	5	7	0.711	
**Central hypoventilation, n (%)**	5	3	2	0.319	
**Pulmonary infection, n (%)**	17	7	10	0.721	
**Hypoproteinemia, n (%)**	10	5	5	0.271	
**Hyponatremia, n (%)**	8	6	2	0.013^*^	9.268 (1.054-81.502), 0.045
**Abnormal brain MRI, n (%)**	19	7	12	1.000	
**CSF tests**					
CSF pressure, median (IQR)	165.00 (129.52-192.50)	155 (140.00-187.50)	170.00 (126.07-202.50)	0.709	
WBC count (n×10^6^), median (IQR)	8.00 (2.00-26.50)	22.0 (8.5-37.00)	3 (2-13.5)	0.004^*^	
CSF protein, n × g/L	376.70 (267.58-550.58)	445.55 (367.7-620.625)	343.85 (234.75-466.75)	0.102	
**Blood tests**					
WBC count, median (IQR), n × 10^9^/L	8.57 (6.80-10.27)	7.90 (5.55-10.83)	8.67 (7.08-10.22)	0.466	
Platelets, median (IQR)	216.00 (178.25-261.50)	206.00 (154.25-279.50)	216 (183.5-260.25)	0.817	
Neutrophils, median (IQR)	5.65 (4.56-7.62)	5.65 (3.50-8.95)	5.64 (4.78-7.14)	0.986	
Lymphocytes, median (IQR)	1.46 (0.85-1.88)	0.83 (0.60-1.48)	1.68 (1.30-2.29)	0.001^*^	0.063 (0.006-0.639), 0.019
Monocytes, median (IQR)	0.58 (0.46-0.75)	0.49 (0.40-0.99)	0.64 (0.48-0.75)	0.345	
**NLR**	3.50 (2.45-8.90)	6.67 (2.95-11.76)	3.17 (2.28-5.81)	0.080	
**MLR**	0.47 (0.28-0.62)	0.60 (0.36-0.75)	0.37 (0.26-0.59)	0.110	
**PLR**	127.60 (88.55-214.68)	217.94 (124.01-312.78)	120.86 (82.07-156.54)	0.018^*^	
**mRS score after immunotherapy, median (IQR)**	1 (1-2)	2 (1.25-2.00)	1 (0.75-2.00)	0.033^*^	
**Hospital stay, median (IQR), days**	62.5 (54.75-65.25)	26.5 (17.25-32.25)	22 (13-32)	0.736	

* indicates P<0.05, OR, odds ratio; CI, confidence interval.

### Laboratory and imaging findings

The initial CSF, brain MRI and laboratory findings are presented in **Table 1**. Lumbar puncture was performed in 33 patients. The CSF intracranial pressure was higher than 180 mmH_2_O in 10 (30.3%) patients, and the CSF WBC count was increased (> 5 × 10^6^/L) in 18 (54.5%) patients. The CSF lymphocyte ratios and total protein were elevated in 27 (81.8%) and 9 (27.3%) patients, respectively. AE-related antibodies, including anti-NMDAR, GABA_B_R, LGI1, α-amino-3-hydroxyl-5-methyl-4-isooxazolpropionic acid receptor (AMPAR1, AMPAR2), and contact protein-associated protein-2 (CASPR2) antibodies, were detected in 18 serum samples and 33 CSF samples. A total of 33 patients were positive for anti-GABA_B_R antibodies in CSF and 17 patients were positive for anti-GABA_B_R antibodies in serum. 33 patients underwent a brain MRI. Of these, 18 (54.5%) exhibited increased signals on T2-weighted or FLAIR images, of which 14 (41.2%) were distributed in the limbic system: 7 patients had bilateral lesions, 6 patients had left-sided lesions, and 2 patients had right-sided lesions (1 patient showed lesions of the right medial temporal lobe and bilateral hippocampus).

### Treatment and follow-up

All patients received first-line treatment, and the median time from onset of the disease to the initiation of immunotherapy was 12.5 (4-186) days. No patients received second-line treatment. In our center, selection of immunotherapy was based on consensus principles. In mild cases, a single first-line immunotherapy was the primary choice. For patients without contraindications, steroids were preferred; otherwise, IVIG was preferred. In patients positive for serum antibodies, PLEX was preferred. For patients with a poor response to monotherapy or severe cases, combined first-line immunotherapy was considered, such as steroids combined with IVIG and/or plasma exchange. First-line immunotherapy could be repeated according to the specific patient status. If patients did not respond well to first-line immunotherapy, second-line immunotherapy was initiated as soon as possible. 12 received steroids (1 g/d for 5 days) alone, 3 received IVIG (0.4 g/kg/d for 5 days) alone, and 1 received PLEX alone. In addition, 18 patients were administered combined first-line immunotherapy: 14 were administered steroids combined with IVIG, 2 were administered steroids combined with PLEX, and 2 were administered steroids combined with IVIG and PLEX. At follow-up, neurological function, relapse, presence of tumors, and mortality were evaluated. The median follow-up time was 22.5 months (0.1-63 months). 7 experienced relapse, with a median time from discharge to relapse of 187 (81-772) days. Additionally, 12 (35.3%) patients had lung cancer: 7 cases were diagnosed at admission, and 6 presented during follow-up. Among these patients with cancer, 6 were confirmed to have SCLC *via* pathological biopsy. All of the patients presented with neurologic symptoms that preceded the diagnosis of cancer. 14 died, with 7 deaths due to lung cancer.

### Predictive factors for poor prognosis of patients with anti-GABA_B_R encephalitis

**Figure 1:**
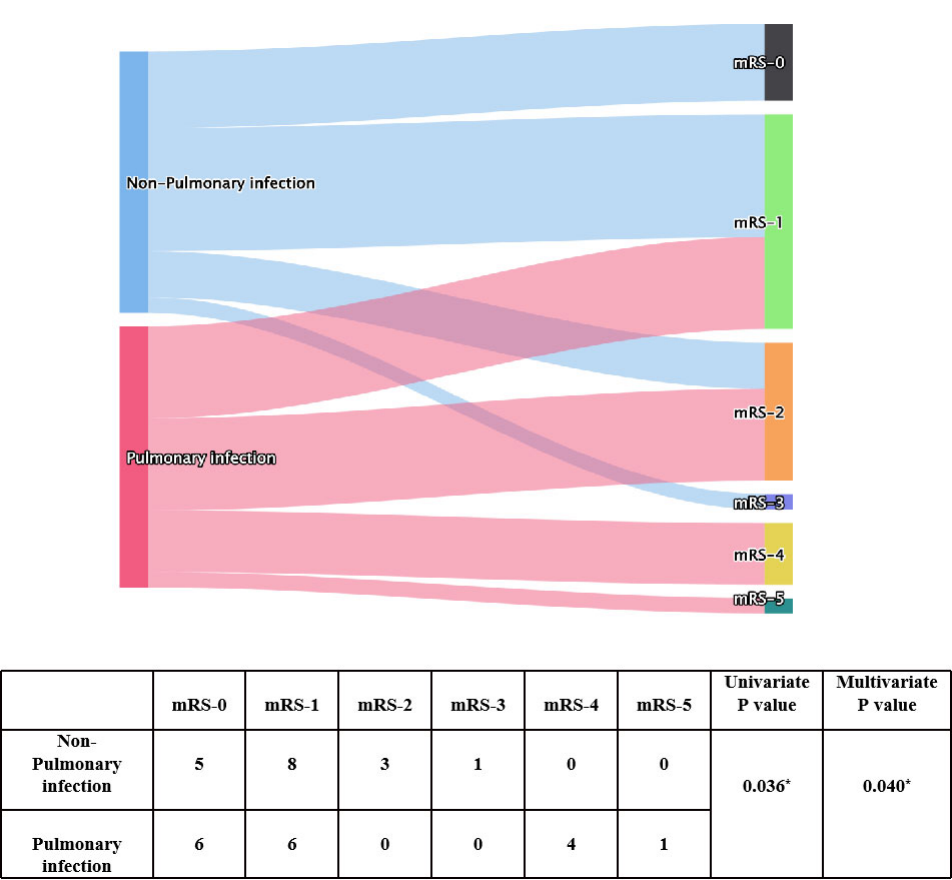
Univariate and multivariate analyses of pulmonary infection presentation and mRS score after immunotherapy. * indicates P<0.05.

**Figure 2:**
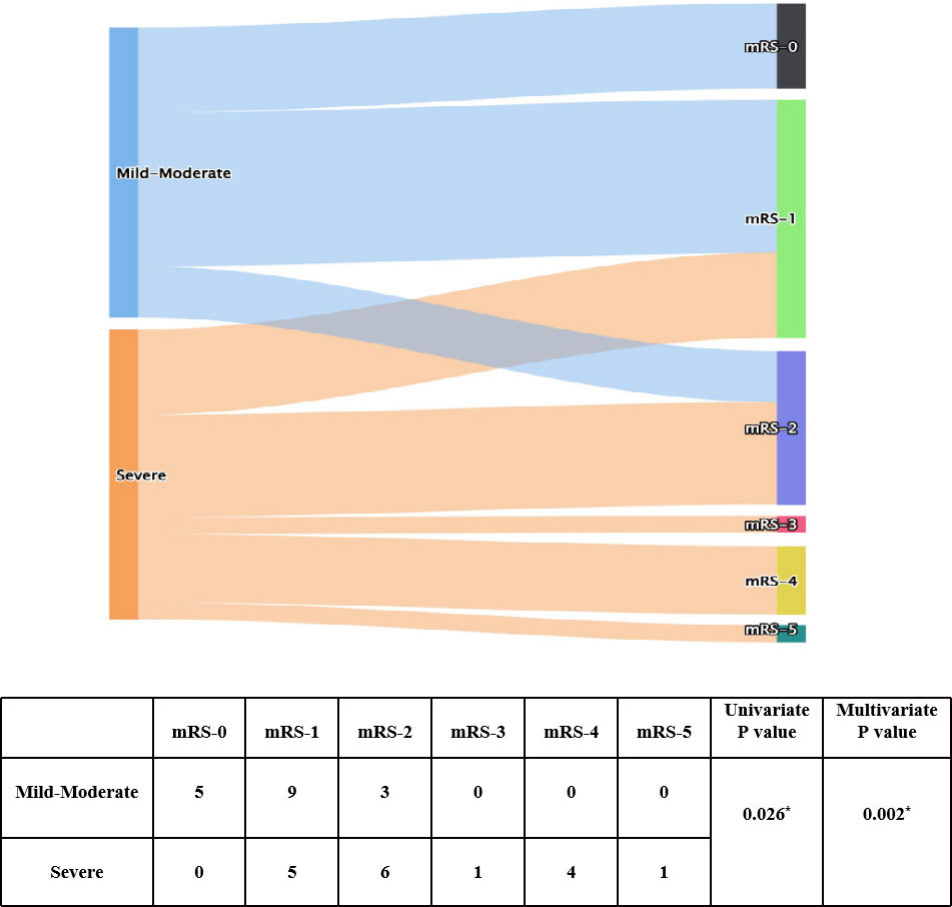
Univariate and ordinal analyses of mRS scores at admission and after immunotherapy. * indicates P<0.05.

To explore factors related to prognosis of patients with anti-GABA_B_R encephalitis, we conducted ordinal regression analysis according to mRS scores (**Table 2**). Univariate analysis indicated baseline mRS scores (P=0.002), psychiatric behavior (P=0.009), hypoproteinemia (P=0.05), pulmonary infection (P=0.036), central hypoventilation (P=0.021), and accompanying tumors (P=0.033) to be associated with significant differences in mRS scores after first-line treatment. All of the above factors were included in the ordinal logistic regression model, and the results showed that pulmonary infection [odds ratio (OR)=17.444, 95% confidence interval (CI): 1.713-177.683, P=0.016] and baseline mRS scores (OR= 17.392, 95% CI: 2.237-135.098, P=0.006) were independent risk factors for failure of first-line treatments in patients with anti-GABA_B_R encephalitis (**Figures 1**, **2**). Moreover, the adjusted ORs of pulmonary infection (OR=9.885, 95% CI: 1.106-88.323, P=0.040) and baseline mRS score (OR= 24.047, 95% CI: 3.294-175.739, P=0.002) were still significant when age and sex were included as covariates in the multiple regression model, further demonstrating the robust predictive value of pulmonary infection and baseline mRS score in anti-GABA_B_R encephalitis therapy.

**Table 2:** Univariate and ordinal regression analysis of predictors for outcomes of anti-GABA_B_R encephalitis immunotherapy.

	Univariate analysis	Ordinal logistics regression			
	P value	Model 1^**^ OR (95% CI)	P value	Model 2^#^ OR (95% CI)	P value
**Sex**	0.975				
**Age**	0.548				
**mRS score at admission**	0.026^*^	17.392 (2.237-135.098)	0.006^*^	24.047 (3.294-175.739)	0.002^*^
**Symptoms**					
Psychiatric behavior	0.009^*^	1.388 (0.120-16.071)	0.793		
Seizures	0.398				
Consciousness declination	0.144				
Cognitive dysfunction	0.561				
Movement disorder	0.111				
Speech dysfunction	0.895				
Sleep disorder	0.152				
Autonomic dysfunction	0.082				
Prodromal symptoms	0.382				
**Tumor presentation**	0.033*	2.737 (0.424-17.655)	0.290		
**ICU admission**	0.144				
**Central hypoventilation**	0.021^*^	3.216 (0.179-57.858)	0.428		
**Pulmonary infection**	0.036^*^	17.444 (1.713-177.683)	0.016^*^	9.885 (1.106-88.323)	0.040^*^
**Hypoproteinemia**	0.050^*^	2.889 (0.271-19.317)	0.447		
**Hyponatremia**	0.177				
**Abnormal MRI**	0.512				
**CSF tests**					
CSF pressure	0.366				
WBC count	0.133				
CSF protein	0.327				
**Blood tests**					
WBC count	0.602				
Platelets	0.188				
Neutrophils	0.512				
Monocytes	0.152				
**NLR**	0.777				
**MLR**	0.487				
**PLR**	0.404				
**Hospital stay**	0.034^*^	1.068 (0.999-1.142)	0.055		

* indicates P<0.05, OR, odds ratio; CI, confidence interval.

**Model 1 included mRS score at admission, hospital stay, Psychiatric behavior, tumor presentation, central hypoventilation, pulmonary infection, and hypoproteinemia.

#Model 2 included all factors from Model 1 plus age and sex.

### Comparisons between the cancer and noncancer groups

To explore whether anti-GABA_B_R encephalitis interacts with cancer, we performed logistic analysis between the cancer and noncancer groups. Univariate analysis indicated significant differences between the group with cancer and the group without cancer with regard to psychiatric behavior (P=0.003), CSF WBC count (P=0.004), ALC (P=0.001), the PLR (P=0.018), and mRS scores (P=0.033) after first-line treatment. All factors with a P value < 0.05 were included in the multivariate logistic regression model. Due to the extreme distribution of psychiatric behavior (all patients in the cancer group had a psychiatric behavior), we performed multivariate logistic regression analysis excluding this variable; we found that ALC (OR: 0.063, 95% CI: 0.006-0.639, P=0.019) and hyponatremia (OR: 9.268, 95% CI: 1.054-81.502, p=0.045) were independent risk factors for anti-GABA_B_R encephalitis accompanied by lung cancer.

## Discussion

In this study, we retrospectively analyzed the clinical features and risk factors for poor prognosis of patients with anti-GABA_B_R encephalitis who received first-line treatment. Moreover, we identified factors related to cases of anti-GABA_B_R encephalitis accompanied by cancer. We found that pulmonary infection and baseline mRS score may be crucial predictors of a poor prognosis in patients with anti-GABA_B_R encephalitis and that low ALC and hyponatremia at the time of admission may predict an underlying risk of developing cancer. However, the NLR, MLR and PLR had no predictive value in terms of the success of first-line treatment.

Of the 34 patients, 26 were male (76.5%), and 8 were female. This result suggests that anti-GABA_B_R encephalitis is more common in males, which is consistent with previous research (6, 21). The median time from onset to admission was 10 days, which is shorter than the 4-week (2–104-week) duration described by Hoftberger B (6). Viral infection is a principal cause of AE (22). However, in our study, only 13 patients (38.2%) exhibited prodromal symptoms of infection, such as fever and headache, indicating that infection was not a trigger for onset in most of our patients.

The GABA_B_R is a G-protein-coupled receptor that belongs to the family of inhibitory synaptic proteins; this family plays an important role in neurotransmitter transmission and synaptic plasticity (23). GABA_B_Rs reduce neuronal activity by inhibiting presynaptic calcium channels and thereby reducing calcium influx. GABA_B_Rs are widely distributed in the CNS and highly localized in the cerebral cortex, hippocampus, cerebellum and thalamus (24). In our study, seizure was the initial symptom of AE in 26 patients (76.5%). In the whole course of the disease, seizure occurred in 30 patients, psychiatric behavior occurred in 23 patients, and cognitive dysfunction occurred in 23 patients, further confirming the above point. Previous studies (25, 26) have verified that anti-GABA_B_R encephalitis should be considered when patients are admitted to the hospital with characteristic manifestations of new-onset seizure or status epilepticus. Seizures may be the major or only clinical symptom of anti-GABA_B_R encephalitis, and approximately 3/4 of patients develop refractory epilepsy (27). In this study, 18(54.5%) showed abnormal inflammation on the T2-weighted FLAIR, which is essentially consistent with the results of Dalmau J (28). Previous studies have found inflammatory changes when analyzing the CSF (29). Although an abnormal MRI is important for diagnosing anti-GABA_B_R encephalitis, lack of MRI abnormalities cannot rule out this disease. Our study further supports this view.

In this study, the baseline mRS score was a crucial predictor for response to first-line treatment. The mRS score was originally developed and validated to assess a patient’s neurological outcome after stroke (30). Later, researchers applied it to assess the severity and prognosis of AE, and, in most studies, patients with AE are divided into groups with a cutoff value of 2. Based on previous research, we assessed the mRS scores of patients with anti-GABA_B_R encephalitis at admission and after first-line treatment. We found that the higher the mRS score at admission was, the higher the mRS score after first-line treatment; that is, the more serious the condition was, the less effective the therapy. The severity of anti-GABA_B_R encephalitis fundamentally reflected the disease-induced inflammation, and the efficacy of treatment largely depended on disease severity, which is consistent with clinical practice. The findings further indicate that patients with a high baseline mRS score should be given more aggressive treatment (combined first-line immunotherapy). Additionally, these results suggest that doctors should give close attention to patients with a high baseline mRS score and communicate with relatives in advance about the possibility of a poor prognosis.

The results of the ordinal analysis showed pulmonary infection is an independent risk factor for failure to response to first-line treatment in patients with anti-GABA_B_R encephalitis. The incidence of pulmonary infection is high in these patients. According to a study by Jingfang Lin, more than two-thirds of anti-GABA_B_R encephalitis patients (18/28, 64.3%) have pneumonia, which is the major cause of short-term mortality (31). In our study, 50% of patients (17/34) developed a pulmonary infection during immunotherapy, and all of them had a worse response to first-line treatments. Additionally, pulmonary infection may be a crucial risk factor for poor prognosis in anti-NMDAR encephalitis (13, 32). The possible reasons are as follows. First, immune dysfunction results in low antibacterial activity of alveolar macrophages. Second, the administration of corticosteroids and immunosuppressants further reduce patient immune function. Third, central hypoventilation might aggravate the infection. Fourth, long-term bedridden status and intubation may increase the risk of pneumonia. In addition, some studies have found that the risk of pulmonary infection is related to the dose of corticosteroids and immunosuppressants: the higher the dose is, the higher the risk of infection (33, 34). All patients in our study received first-line treatment: 12 received steroids, 14 received steroids combined with IVIG, 2 received steroids combined with PLEX, and 2 received steroids combined with IVIG and PLEX. To treat this condition, patients are administered high doses of corticosteroids for long durations. Moreover, pulmonary infection in patients with immune dysfunction differs from that in patients with normal immune function because of the increased risks of opportunistic infections and severe bacterial infections. Therefore, close attention should be devoted to the occurrence of pulmonary infections in patients with anti-GABA_B_R encephalitis. In the present study, all patients were assessed for the risk of pneumonia before immunotherapy and regularly over the course of immunotherapy. In addition to a CT scan of the thorax, we also recommend examination of pathogens. If pneumonia developed, we immediately initiated anti-infective therapy. Mild pneumonia had little influence on immunotherapy; however, in cases of definite severe infection, IVIG was given priority, and the use of steroids was discontinued until the infection was controlled. Overall, appropriate prophylactic measures and aggressive therapy for pulmonary infection might help to improve patient prognosis.

Previous studies (28) have reported that approximately 50% of patients with anti-GABA_B_R encephalitis harbor an underlying cancer, particularly SCLC. The pathogenesis of cancer is related to abnormalities in the immune system. In this study, 12 (35.3%) were complicated with lung cancer, with 6 confirmed to have SCLC. The lower incidence of cancer in this study may be related to the short follow-up time. Once patients are diagnosed with anti-GABA_B_R encephalitis, cancer screening (especially for lung cancer) should be initiated as soon as possible. If the first cancer screening is negative, regular follow-up screening should be implemented. Additionally, screening is recommended at 3–6 months after discharge and then once a year for at least 4 years (35). In our univariate analysis, lung cancer was indicated to result in significant differences in mRS scores after first-line treatment (P=0.033). However, in the ordinal logistic regression model, the influence of lung cancer was not significant. In this study, our purpose was to find out the potential factors that affect the response to first-line treatment rather than survival in patients with anti-GABA_B_R encephalitis. Therefore, it is worthwhile to explore if the presence of lung cancer affecting the survival in a larger cohorts. This result might have been due to the small sample size of our study, which is a limitation. In the future, larger study cohorts are needed to confirm this hypothesis. Moreover, we found that a lower ALC might be a predictor of anti-GABA_B_R encephalitis accompanied by lung cancer. Normally, lymphocyte subpopulations maintain a dynamic balance to ensure stable immune function. The immune system, especially the strength of cellular immune function, is an important intrinsic protective factor against cancer occurrence. In recent years, many important studies have shown that the strength of the immune system is strongly related to the aggressiveness and prognosis of cancer. ALC represents the strength of the immune system and is an independent factor that influences cancer prognosis. In general, lymphocytes inhibit the proliferation of malignant cells in the body (36). In this study, patients with anti-GABA_B_R encephalitis and reduced ALC had a higher incidence of lung cancer, similar to the findings of a previous study. As the present study was retrospective in nature, lymphocyte subsets were not evaluated, and the specific mechanism underlying this relationship needs to be clarified.

Recent studies have found that the NLR, MLR and PLR, which are new biomarkers of inflammation (15), can stably reflect the body’s inflammatory state and correlate with classic inflammatory mediators [such as levels of C-reactive protein (CRP), interleukin-6 (IL-6) and tumor necrosis factor alpha (TNFa)]. The NLR serves as a biomarker of systemic inflammation in systemic lupus erythematosus (37), ulcerative colitis (38), and rheumatoid arthritis (39). Furthermore, some studies have suggested that the NLR is related to the severity, treatment and prognosis of CNS autoimmune diseases, such as multiple sclerosis (40) and AE (12, 13). In this study, we found no correlations of the NLR, MLR, or PLR with mRS scores after first-line treatment for anti-GABA_B_R encephalitis. James Broadley et al. (14) showed that a high NLR is associated with first-line treatment failure but that a high MLR was not associated with AE prognosis, consistent with our previous research on the MLR. We also utilized the PLR for the first time in the present study but found that it did not affect prognosis. Differences in the effects of the NLR on prognosis may be due to differences among study cohorts. Anti-GABA_B_R encephalitis is a type of AE mediated by neuronal cell surface antibodies, which are currently believed to be largely moderated by humoral immunity, but the exact pathological mechanisms of immune proliferation and transmission remain unclear (28). The NLR, MLR and PLR may be more closely related to encephalitis mediated by intracellular antibodies rather than neuronal cell surface antibodies; the former are considered to be cellular immune responses mediated mainly by T cells and pathology is characterized by a large number of infiltrating macrophages and microglia (14). The relationships between peripheral inflammatory indicators and AE prognosis require further multicenter studies with larger sample sizes.

In summary, our study had several limitations. First, this study had a retrospective design. Second, although we applied strict inclusion criteria, the sample size at our single center was still relatively small due to the low incidence of anti-GABA_B_R encephalitis in the general population. In the future, multicenter prospective studies are needed to confirm our results.

## Conclusions

To date, studies have yet to identify the exact clinical characteristics that predict poor prognosis of patients with anti-GABA_B_R encephalitis. This study demonstrates that pulmonary infection and baseline mRS scores were independent risk factors for a poor prognosis of patients with anti-GABA_B_R encephalitis after first-line treatment. Moreover, ALC and hyponatremia might be potential biomarkers in the clinical evaluation of patients with anti-GABA_B_R encephalitis accompanied by lung cancer.

## Data availability statement

The original contributions presented in the study are included in the article/supplementary material. Further inquiries can be directed to the corresponding author.

## Ethics statement

The experiments involving human participants were reviewed and approved by the Ethics Committee of the First Affiliated Hospital of Zhengzhou University. The patients/participants provided written informed consent prior to participation in this study.

## Author contributions

JD and YL designed the research. DX, JLv performed the research and data analysis. MT and JHL collected the data. JD wrote the paper; and JD, DX, JLv, TW and JHL critically revised the paper. All authors contributed to the article and approved the submitted version.

## Conflict of interest

The authors declare that the research was conducted in the absence of any commercial or financial relationships that could be construed as a potential conflict of interest.

## Publisher’s note

All claims expressed in this article are solely those of the authors and do not necessarily represent those of their affiliated organizations, or those of the publisher, the editors and the reviewers. Any product that may be evaluated in this article, or claim that may be made by its manufacturer, is not guaranteed or endorsed by the publisher.
